# Supplementary material for: Structural exposure of different microtubule binding domains determines the propagation and toxicity of pathogenic tau conformers in Alzheimer’s disease
Source: PLoS Pathog. 2025 Jun 13;21(6):e1012926. doi: 10.1371/journal.ppat.1012926 (PMC12187016; doi:10.1371/journal.ppat.1012926)
Supplement: S1 Table — Demographics of AD cases, disease duration (DD), neuropathological classification of AD stage, postmortem intervals (PMI), ApoE alleles, and conformation dependent assay (CDI) data on Amyloid beta 42 (Ab42) and detergent-insoluble TAU in the cortex. (DOCX) [file ppat.1012926.s007.docx]

**S1 Table**. Case information for all samples used in the study. Demographics of AD cases, disease duration (DD), neuropathological classification of AD stage, postmortem intervals (PMI), ApoE alleles, and conformation dependent assay (CDI) data on Amyloid beta 42 (Ab42) and detergent-insoluble TAU in the cortex.
