# Supplementary material for: Structural exposure of different microtubule binding domains determines the propagation and toxicity of pathogenic tau conformers in Alzheimer’s disease
Source: PLoS Pathog. 2025 Jun 13;21(6):e1012926. doi: 10.1371/journal.ppat.1012926 (PMC12187016; doi:10.1371/journal.ppat.1012926)
Supplement: S4 Table — (DOCX) [file ppat.1012926.s010.docx]

**S4 Table**. Summary data on primary mouse neuron cultures and neuronally differentiated SH-SY5Y cells exposed to AD brain-derived tau conformers.
