# Supplementary material for: Structural exposure of different microtubule binding domains determines the propagation and toxicity of pathogenic tau conformers in Alzheimer’s disease
Source: PLoS Pathog. 2025 Jun 13;21(6):e1012926. doi: 10.1371/journal.ppat.1012926 (PMC12187016; doi:10.1371/journal.ppat.1012926)
Supplement: Attachment — Submitted filename: PPPATHOGENS-D-25-00167_RESPONSE_TO_REVIEWERS.docx [file ppat.1012926.s012.docx]

­­­­­­­­­­­Dear Editors,

We thank the Editors and all Reviewers for their thoughtful comments and for the opportunity to clarify and improve the aspects of our paper. Our responses are as follows:

**Reviewer #1:**

1. Why was recombinant tau (rectau441) used in the hydroxylation decay experiments as opposed to a control (healthy) brain homogenate? Control brains of age matched patients likely contain tau aggregates that never progress to AD and would perhaps produce a more suitable comparison.

***Response***

*Reviewer #1’s comment is well taken, and we have inserted the following explanation in the Results section:*

*“We used as a hydroxylation control recombinant tau441 (green line in* ***Fig. S1B, C, D, E, F****) instead of the alternative non-neurological, age-matched human brain because the reaction is quenched by a large excess of other proteins and Fe^++^ in human brain homogenates. Both normal monomeric brain tau and recTau441 (****Fig. 1****) have open (unstructured) conformations [**49**]; if misfolded tau is present in age-matched non-neurological brain homogenates, it is not detectable with our Europium-labeled antibodies [**3**]. Under the conditions optimized for the differentiation of conformers of misfolded tau, the hydroxylation rate of tau441 monomers was 30-200-fold faster than that of insoluble aggregates of AD brain-derived tau, and all epitopes were fully hydroxylated in the first two minutes (green line in* ***Fig. S1B, C, D, E, F****). Rapid hydroxylation of tau441 monomers requires hydroxyl radical exposure on the millisecond time scale and a fully automated synchrotron hydroxylation station (****Fig. S3B****).”*

2. For the RT-QuIC assays, there is no mention of a reducing agent in the RT-QuIC buffer, how is K18 reduced? Also, in most published tau RT-QuIC assays seeding in control brains has been demonstrated. Does the K18 assay as presented in this work show any seeding activity of control brains at 10-4? Furthermore, a full dilution series for at least one AD brain should be presented to indicate that the seed is being diluted out.

***Response***

*We appreciate Reviewer #1’s comment. The rectau441 was reduced by 2mM DTT and the detailed description of E. coli expression, purification, RT QuIC assay with K18 and K19 tau substrates and serial dilutions of AD, FTD, Pick’s disease, and normal brain homogenates has been validated and published recently in our 2022 Sci Transl Med paper[**3**]. In addition, our protocols for the induction of tau pathology in mouse neurons[**28**] and neuronal differentiation of human SH-SY5Y cells have been previously validated [**56**].*

*We have inserted the following explanation in the Results section:*

*“To collect a representative spectrum of detergent-insoluble, fibrillar and nonfibrillar, tau aggregates accumulating in the AD brain, we adopted the protocol used successfully in purification of human prions [**36**,**47**]. This protocol minimizes the nonspecific effects of human brain lysate components without proteases and allows us to investigate the same misfolded tau conformers in four independent systems simultaneously: (i) hydroxylation footprinting, (ii) RT QuIC for seeding potency[**3**], (iii) induction of tau pathology in mouse neurons[**28**], and (iv) induction of tau pathology and calcium flux in human neuronally differentiated SH-SY5Y cells[**56**]. Apart from the hydroxylation protocol, our tau-seeding protocols have been previously validated [**3**,**28**,**56**]. Atomic force microscopy (AFM) showed morphological heterogeneity of preparations with variable contents of paired helical filaments (PHs)[**57**], and smaller aggregates (****Fig S2A, B, C, D****), and the overlapping conformational stability assay (CSA)[**3**,**58**] profiles of misfolded tau demonstrated that the final sample used in the hydroxylation profile protocol is representative of the tau conformers present in the brain homogenate (****Fig. S2E****). SDS PAGE showed expected heterogeneity [**59**] with a variable fraction of SDS-resistant aggregates and oligomers, and an excess of four repeats (4R) over three repeat (3R) tau isoforms (****Fig. S2F****) previously reported in the hippocampal cortex [**3**].*

3. It is fascinating that R4 was most variably protected (359-373), and the C-tail (368-441) was the least despite the slight overlap in sequence. Does the Fitzpatrick cryo-EM structure of AD or any of the Scheres cryo-EM structures provide any insight as to why these residues may be important in driving seed potency?

***Response***

*We agree with the reviewer that the differences between R4 and C-tail are striking. Our samples were mixtures of fibrillar and non-fibrillar tau aggregates (****Fig. S2A, B, C, D****) present in AD brain homogenates [**3**]. We expanded the Discussion section as follows:*

*“New evidence that cryo-EM structures of prions[**79**], and tau filaments[**20**,**22**,**70**], isolated from animal and human brain are impacted by PTMs and retain auxiliary, non-proteinaceous densities suggests that such associations are more broadly maintained across different filament extraction protocols from the human brain and play a role in their formation and interactions. To assign the structural interplay between PTMs and auxiliary noncovalent interactors to different domains[**78**] in fibrillar and nonfibrillar assemblies will require separation of different aggregates by sucrose gradients or HPLC integrated with synchrotron hydroxylation and mass spectrometry to monitor high resolution structure and PTMs in different tau domains, and then correlating the data with cryo-EM.”*

**Reviewer #2:**

**Major Points**

1. The authors appear to infer causality from association. That is, they find heterogeneity in tau assembly structures and seeding activity associated (but not overwhelmingly so) with brain homogenates from AD cases of distinct phenotype. On its own these measures are associational, but reflect causality.

***Response***

*To address the reviewer’s concern, we have expanded the Results section as follows:*

*“To collect a representative spectrum of detergent-insoluble, fibrillar and nonfibrillar, tau aggregates accumulating in the AD brain, we adopted the protocol used successfully in purification of human prions [**36**,**47**]. This protocol minimizes the nonspecific effects of human brain lysate components without proteases and allows us to investigate the same misfolded tau conformers in four independent systems simultaneously: (i) hydroxylation footprinting, (ii) RT QuIC for seeding potency[**3**], (iii) induction of tau pathology in mouse neurons[**28**], and (iv) induction of tau pathology and calcium flux in human neuronally differentiated SH-SY5Y cells[**56**]. Apart from the hydroxylation protocol, our tau-seeding protocols have been previously validated [**3**,**28**,**56**]. Atomic force microscopy (AFM) showed morphological heterogeneity of preparations with variable contents of paired helical filaments (PHFs) [**57**] and smaller aggregates (****Fig S2A, B, C, D****), and the overlapping conformational stability assay (CSA)[**3**,**58**] profiles of misfolded tau demonstrated that the final sample used in the hydroxylation profile protocol is representative of the tau conformers present in the brain homogenate (****Fig. S2E****). SDS PAGE showed expected heterogeneity [**59**] with a variable fraction of SDS-resistant aggregates and oligomers, and an excess of four repeats (4R) over three repeat (3R) tau isoforms (****Fig. S2F****) previously reported in the hippocampal cortex [**3**].”*

2. Assumptions about structure/conformation are based on solvent accessibility of specific domains. This method is generally acceptable in the setting of purified forms of protein, where it is unlikely that other factors are not present in the complexes studied. In this case, however, the authors are studying tau within brain homogenates. How can the exclude the possibility that any differences they see are not due to associated factors bound to and masking components of otherwise identical assemblies? This could easily confound their data.

***Response***

*We interpreted the hydroxylation epitope data conservatively as a structural “exposure” that “implicate” or “determine” the differential effects of AD tau conformers and we did not state the direct structural causality in the text, title, or in the abstract. We also stated the following in the original discussion:*

*“Whether differences in conformation drive distinct PTMs, or vice versa, is an important question not only in AD but also in other tauopathies, including progressive supranuclear palsy (PSP), frontotemporal dementia (FTD), and Pick’s disease. Integrating photochemical hydroxylation footprinting with mass spectrometry of tau in future experiments will address this question by allowing the simultaneous investigation of both aspects. These approaches are applicable to both filamentous and oligomeric structures and should also answer the question of whether the differences in hydroxylation footprinting a result of conformation and particle size are solely or whether they are modified by Sarkosyl-resistant ligands or attached lipids.”*

*We have expanded the Discussion section as follows:*

*“New evidence that cryo-EM structures of prions[**79**], and tau filaments[**20**,**22**,**70**], derived from animal and human brain are impacted by PTMs and retain auxiliary, non-proteinaceous densities suggests that such associations are more broadly maintained across different filament extraction protocols from the human brain and play a role in their formation and interactions. To assign the structural interplay between PTMs and auxiliary noncovalent interactors to different domains[**78**] in fibrillar and nonfibrillar assemblies will require separation of different aggregates by sucrose gradients or HPLC integrated with synchrotron hydroxylation and mass spectrometry to monitor high resolution structure and PTMs in different tau domains, and then correlating the data with cryo-EM.”*

3. Orthogonal approaches to study structures are needed. Cryo-EM (even 2D reconstructions) or some orthogonal approach to compare seed structure (e.g. recently published Vaquer-Alicea et al. Sci Adv. 2025) would help solidify their findings. Otherwise, we are left with limitations raised in point (2) above.

***Response***

*We have inserted the response statement in the Discussion section (point 2).*

4. Resolve patterns within defined assemblies. Failing orthogonal validation (much preferred), it would also be useful to extract soluble tau, resolve the oligomers by SEC (or even look at soluble tau overall) and perform the OH labeling. By restricting their analyses to insoluble tau they are potentially biasing the analyses.

***Response***

*The reviewers’ comments suggest future research directions generated by our bench-formatted photochemical hydroxylation introduced for rapid differential screening of tau aggregates in this study, and we discussed them in the Discussion section and in the response to points 1 and 2. Moreover, the resolution of the current protocol is limited to the amino acid sequence and structural* ***exposure*** *of the antibody epitope. To assign the differences to fibrillar and nonfibrillar assemblies and the interplay between* ***structure,*** *PTMs, and noncovalent auxiliary molecules in distinct domains will require separation of different aggregates by sucrose gradients or HPLC integrated with high-resolution synchrotron hydroxylation monitored by mass spectrometry, followed by correlation of the data with cryo-EM. We have expanded the Discussion section in response to point 2.*

5. On its own, Figure 2 is difficult to interpret in terms of significance. Data underlying the figure should be clearly shown. It appears data derive from one case (according to figure legend). How is this significant?

***Response***

*We described the protocol in detail in the Methods section — each epitope half-life was generated by fitting six time points, each run in duplicates; the mean and errors of the fits are shown in* ***Table S2,*** *and the immunoassay has a coefficient of variation (100*S.E.M./Mean) ≤7% [**3**,**25**].*

*We revised the statement in the Results and* ***Fig. 2A, B*** *legends:*

*“Within the spectrum of Alzheimer’s disease phenotypes, the rapidly progressive subtype is characterized by rapid cognitive decline [**4**,**7**,**12**], a higher frequency of missense variants in known AD risk genes[**1**], a low frequency of the e4 allele of the APOE gene[**4**], and an accumulation of amyloid beta and misfolded tau proteins with distinct conformational characteristics [**3**,**28**,**51**] and interactomes[**7**,**14**,**15**]. Using the consensus criteria of more than six Mini-Mental State Examination (MMSE) points per year or death within 3 years of initial neurological diagnosis of dementia [**1**,**4**,**7**,**12**,**61**], the data indicated that the R4 domain was more protected from hydroxylation in a number of cases with longer disease durations (****Fig. 2A, B****). However, this trend was not statistically significant due to the large data variance in each group. Notably, the variance in half-life observed in the R4 domain exceeded the C-terminal tail variance by ~9-fold. We interpreted the data as evidence of a distinct pattern of MTBD epitope exposure in individual AD tau conformers, implicating major inter-individual differences in the structural organization of the MTBD in AD brain-derived tau. Within the distinct patterns of protection in each of the conformer domains, photochemical footprinting provided direct evidence for the major variability in the fourth repeat (R4) of MTBD.“*

6. Cell death data in Fig. 4b is weak and unconvincing. This type of assay can’t reliably be used to indicate differences in tau assembly conformation, as it is going to be highly susceptible to artifacts based on differences in disease state between individuals (e.g. degree of insoluble material).

***Response***

*We inserted following response to the Results section:*

*“The differential cell impacts of human AD brain-derived insoluble tau at the same concentration determined after complete denaturation with 4M Gdn HCl by conformation-dependent immunoassay (CDI) indicate distinct structural organization of each isolate and interactome epitopes. Using the same sample in two different cell systems expressing full length mice and homologous human tau implicate structural exposure of the R4 domain in seeding and propagation and thus confirm the in vitro seeding data obtained with K18 tau fragments in RT QuIC.”*

7. Unclear what is the point overall of Figs. 4, 5. It is well known that AD lysates have more seeding activity vs. controls. Therefore, it is unclear why these figures are included, and how they advance the thesis of the paper.

***Response***

*We believe that we have addressed this comment in point 6.*

8. Variance in assays must be established. The authors make a great deal of variance in Gdn denaturation (Fig. 1), but it is unclear what is the variance in this assay with samples of relatively similar pathology. That is, just because there is variance in the assay, it doesn’t mean that there are different conformations in the cases—the assay itself may be quite variable. They must provide “calibration” cases to show the denaturation curves are meaningful.

***Response***

*We have inserted the following in the Results section:*

*“The conformational stability assay (CSA) was derived from previously validated protocols for differentiating distinct conformers of human and animal prions, amyloid beta, and tau [**4**,**36-46**] and data in the frontal cortex (****Fig. 1B*** *) correspond to the variable conformational stability of AD brain-derived tau aggregates we reported in the hippocampal cortex previously [**3**].”*

**Reviewer #3:** Text - In general the text reads well and intriguing points. However, the paper needs more controls in critical areas to support the authors claims.

Major Points:

1. The authors report that “The enrichment protocol for AD brain-derived insoluble tau was adopted from the purification of distinct strains of prions without proteases [36,38] and was designed to maintain full-length tau and remove noncovalent interactors”, however there is no data showing that noncovalent interactors have been removed. For the prion protein proteases like PK remove many of the non-protease resistant proteins helping to remove non-prion protein, however, even with multiple rounds of protease treatment, PTA precipitation, and resuspension small amounts of non-prion proteins, or their degradations products, persist. The authors should show blots that indicate that non-covalent interactors have been removed.

***Response***

*We have responded to the similar concern of Reviewer 2 and expanded the Results section as follows:*

*“To collect a representative spectrum of detergent-insoluble, fibrillar and nonfibrillar, tau aggregates accumulating in the AD brain, we adopted the protocol used successfully in purification of human prions [**36**,**47**]. This protocol minimizes the nonspecific effects of human brain lysate components without proteases and allows us to investigate the same misfolded tau conformers in four independent systems simultaneously: (i) hydroxylation footprinting, (ii) RT QuIC for seeding potency[**3**], (iii) induction of tau pathology in mouse neurons[**28**], and (iv) induction of tau pathology and calcium flux in human neuronally differentiated SH-SY5Y cells[**56**]. Apart from the hydroxylation protocol, our tau-seeding protocols have been previously validated [**3**,**28**,**56**]. Atomic force microscopy (AFM) showed morphological heterogeneity of preparations with variable contents of paired helical filaments (PHFs) [**57**] and smaller aggregates (****Fig S2A, B, C, D****), and the overlapping conformational stability assay (CSA)[**3**,**58**] profiles of misfolded tau demonstrated that the final sample used in the hydroxylation profile protocol is representative of the tau conformers present in the brain homogenate (****Fig. S2E****). SDS PAGE showed expected heterogeneity [**59**] with a variable fraction of SDS-resistant aggregates and oligomers, and an excess of four repeats (4R) over three repeat (3R) tau isoforms (****Fig. S2F****) previously reported in the hippocampal cortex [**3**].”*

*We expanded the Discussion section with following:*

*“New evidence that cryo-EM structures of prions[**79**], and tau filaments[**20**,**22**,**70**], derived from animal and human brain are impacted by PTMs and retain auxiliary, non-proteinaceous densities suggests that such associations are more broadly maintained across different filament extraction protocols from the human brain and play a role in their formation and interactions. To assign the structural interplay between PTMs and auxiliary noncovalent interactors to different domains[**78**] in fibrillar and nonfibrillar assemblies will require separation of different aggregates by sucrose gradients or HPLC integrated with synchrotron hydroxylation and mass spectrometry to monitor high resolution structure and PTMs in different tau domains, and then correlating the data with cryo-EM.”*

2. The authors show in supplemental Figure 3 that the amount of tau detectable by western blot decreases over time, which is equated to the loss of the epitope to which the antibody binds. However, the isolated tau in the blots is much larger than the purified tau, indicating that the material on the blots is not denatured fully and retains its aggregated structure while being run over a gel. If preparation for blots is unable to break up the larger aggregates of tau then it is possible that the apparent loss of tau on the blots may do not be due to hydroxyl damage to antibody epitopes and maybe the result of changes in the aggregate structure that exclude antibody interaction. Another possibility is that hydroxyl treatment induces further aggregation of tau to the point that it can no longer enter the gel. The authors should demonstrate that the change in tau signal in the western blots is not the result of more aggregation with a sedimentation assay to evaluate aggregation state, a dot blot that captures aggregates off all sizes, or by showing a Coomassie stain or silver stain of the gels associated with these blots, which would have to be a repeat of the experiment unless the authors have this data on hand.

***Response***

*The comment is well taken, and we expanded the Results section to the following:*

*“Mass spectrometry (MS) monitoring of footprinting performed at high concentrations of hydroxyl radicals generated in synchrotrons does not indicate significant protein crosslinking [**29**,**31**,**32**,**47**,**50-55**]. Additionally, our experiments with monomeric recombinant tau441 in the open conformation indicated progressive loss of antibody affinity on WBs with no concurrent crosslinking of tau monomers in silver staining (****Fig. S3B****). We did not observe detectable concentrations of cross-linked peptides by MS in recTau441 monomers or AD brain-derived aggregates (****Fig. S1G****)”.*

3. It is difficult to evaluate the calcium signaling experiments with the Figure being incomplete.

***Response***

*We have uploaded the revised Figures and corresponding legends.*

4. In supplemental Figure 1 G the region encompassing R4, 337 to 369, shows similar hydroxylation between AD1, AD2, and recombinant Tau, though it is difficult to make statistical arguments with only duplicated samples. With all samples showing similar hydroxylation is hydroxylation at R4 actually a good site for monitoring structural/conformational properties in tau that dictate conversion rates? The authors should comment on this aspect of their data.

***Response***

*We have revised the description of* ***Fig. S1G*** *and inserted into the Results section as follows:*

*“To validate the antibody epitope decay data, we used the mass spectrometry (MS) after a single 8 min hydroxylation step and monitored the exposure or protection of amino acid side chains within the antibody epitopes (****Fig. S1G****). The MS provided an independent readout in two representative AD cases and rectau441 at single amino acid resolution and demonstrated corresponding hydroxylation differences for amino acid side chains within antibody epitopes (****Fig. S1B, C, D, E, F****). Coefficient of variation (100 × SEM)/Mean) of duplicate footprinting assay is ≤7%.”*

***Response***

Figures - While the text is well written and to the point, the figures need an overhaul. As they currently stand the figures are inconsistent and difficult to follow.

Major Points:
1. Figure 5 panel F is missing.

***Panel F added to new Fig. 5.***

2. Figure 5B is missing labels on the x-axis and the line for the y-axis. The y-axis is also missing its label.

***Corrected.***

3. Supplemental Figure 3 appears to be pixelated, and authors should check the dpi of gel images.

***Corrected***.

4. Text on images in Figure 3D is pixilated and should be converted into a vector formation. The image dpi appears to be fine but the distortions on the text make it difficult to read.

***Corrected.***

5. Text on images in Figure 4F is pixilated and should be converted into a vector formation. The images also appear a bit fuzzy and readers would benefit form having higher quality images to look at.

***Corrected.***

6. Error bars are cut off in supplemental Figure 2.

***Corrected.***

7. It is not visually clear what the significance indicator “*” in Figure 4B refers to. Please consider using lines to make it visually more clear which data sets are significantly different as was done in panel E.

***Corrected.***

8. Many Figures use data without labels, and this creates confusion. Figure 3A has many lines with different colors and it is not clear what each line goes to. If it is simply showing the range seeding times then that should be made clear, however, the Figure legend and text give the impression that these lines may show both test samples and controls. Differences in samples should be indicted with clear color distinctions.

***Corrected.***

9. Figure 3D has a critical scaling issue. Does the dashed line box inset in the larger image represent the lower image exactly? If so there is a problem with the scale bars. The smaller box is roughly one third of the width of the image, yet the scale bar is only different by about 1.25 x.

***Corrected.***

**Part III – Minor Issues**: Editorial and Data Presentation Modifications
Please use this section for editorial suggestions as well as relatively minor modifications of existing data that would enhance clarity.

Reviewer #1: 1) Abstract, last sentence change “ti”, to “it”.

***Corrected.***

Reviewer #2: (No Response)

Reviewer #3: Text - While many of the minor points listed below are easily fixable with changes in wording to the text, some of the points may be more critical points depending upon how the authors address them.

Minor Points:
1. Figure legends use capitalization and acronyms inconsistently. Figure 3 legend both “thioflavin” and “Thioflavin” in the same sentence.

***Corrected.***

2. Spacing appears inconsistent in reference brackets. See “K18 (4R) tau substrates [44,45 ] (Fig. 3a” and compare to “AD cases with faster clinical progression [3]”

***Corrected***

3. The authors report “SDS-insoluble high-mass oligomers of tau and a limited ~20-fold dynamic range precluded quantitative evaluation (Fig. S3)”. Is the material shown in Figure S3 the insoluble mass? If so, is there material that was not able to make it into gels because it could not be denatured at all in sample buffer?

***Response***

*The AD brain-derived tau aggregates are highly resistant to SDS, and with the contribution of numerous and variable posttranslational modifications, the result is smeared with a molecular mass ladder (****Fig. S2 and Fig. S3****, see for comparison [**59**]), in contrast to the highly selective extraction protocols used for PHFs in cryo-EM [**20**,**70**,**106**,**107**]. To completely dissociate and denature tau oligomers and aggregates would require denaturation with Gdn HCl we used for our immunoassays, but because Gdn HCl is causing SDS precipitation, it is incompatible with SDS-PAGE.*

4. The authors indicated that the R4 domain is the most “reliable predictor of seeding activity” as the other domains show poor correlations. However, the R value is ~0.39 for R4, which is not a very impressive R value despite the significance of the fit being 0.0003. The authors should comment on this discrepancy and what the low R value means for the interpretation of their data / models.

***Response***

*The R values are impacted by high data variance we discussed in the comments to the hydroxylation data in* ***Fig. 2****. The data were analyzed with linear and nonlinear regression models using SPSS 28 software, and the P values obtained for the models and with ANOVA are consistent.*

5. The difference in the R and P values for R4 and R3/4 are minimal. What is the cutoff that justifies R4 being acceptable to use but not R3/4?

***Response***

*The point is well taken, and we expanded the Results section with following:*

*“The regression analyses of in vitro RT QuIC seeding and hydroxylation data (****Table 2****) may suggest also significant contribution of R3 domain because the epitope of mAb 77G7 covers both R4 (19 amino acids) and R3 (16 amino acids) domains, but to discern the contribution of R3 domain will require high resolution synchrotron hydroxylation footprinting monitored with mass spectrometry. Nevertheless, the seeding data in mice neuronal cultures (****Fig. 3E****) and neuronally differentiated human cells (****Fig. 5D****) consistently implicate R4, not R3 domain, as the significant driver of seeding in vivo.”*

6. The authors should add speculation on why the other domains tested have such a high variability compared to R4.

***Response***

*The responses to the analogous comments of Reviewers #1 and #2 were inserted to the Discussion.*

7. Supplemental Figure 1 G is difficult to read, and it is hard to compare stretches of amino acids due to the way data is shown. This Figure is a critical support for their work that should be reworked to make it easier to interpret.

***Response***

*See below point 8.*

8. The data in supplemental Figure 1 G takes and SEM of duplicate samples. Error analysis on duplicated samples is controversial and Figures should show individual points with trends or add a third sample.

***Response:***

*As suggested, we revised the* ***Fig. S1G*** *and denoted the amino acid positions in the MTBDs on x axis of MS data, replotted the* ***Fig. S1B, C, D, E, F*** *with individual data points of duplicate assays, and expanded the Results section:*

“*To validate the antibody epitope decay data, we used the mass spectrometry (MS) after a single 8min hydroxylation step and monitored the exposure or protection of amino acid side chains within the antibody epitopes (****Fig. S1G****). The MS provided independent readout in two representative AD cases and rectau441 at single amino acid resolution and demonstrated corresponding hydroxylation differences for aminoacid side chains within antibody epitopes (****Fig. S1B, C, D, E, F****). The coefficient of variation (100*S.E.M./Mean) of duplicate footprinting assay is ≤7%.”*

Figures
Minor Points:
1. Graphics in Figures 1 and 4 appear pixelated and should be recreated with a higher dpi image or replaced with vector graphics.

***Corrected.***

2. Text overlaps in non-conventional ways in Figure 5 A. The “/” overlaps with the “)”. This is likely a formatting / graphical issue and should be fixed.

***Corrected***.

3. Text in Figures have inconsistent spacing. To be specific single and double spacing are used inconsistently.

***Corrected***.

4. The text in supplemental Figure 3 that sits on gel images appears stretched.

***Corrected.***

5. Supplemental Figure 5C appears to be a pixilated image as opposed to a vector graphic. Please either upload a higher quality image or convert to a vector graphic.

***Corrected.***

6. Spacing of panels is inconsistent between Figures and the content of individual Figure sections often overlap into other figure sections creating confusing layouts that are hard to follow. Please clean up figure spacing.

***Corrected.***

7. Some images are too dark to properly see immunofluorescence staining and should be brightened or the image quality should be enhanced.

***Corrected.***

8. Figure 2 inconsistently uses white boxes behind text. Numbers for half-life have white boxes behind them which makes them easy to ready but other numbers do not, “269-281”. The text without boxes are hard to read and the cut into Figure graphics altering their appearance. Data in plots also cuts through numbers making it hard to read the numbers. In the upper panel, orange data lines, the number “10” is partially cut off by an invisible box and the text box behind the number has white borders that create a distortion on the plot. Plot layout, numbers, and data graphics overlap in inconsistent ways with some obscuring lines and numbers. This should be fixed.

***Corrected.***

9. The usage of different colored text to indicate different proteins, such as TuJ1 and Tau, is a great idea but the color scheme used will make it difficult to distinguish for some readers. TuJ1 and Actin text color in supplemental Figure 5 were particularly difficult to discern for me.

***Revised.***

10. Through Figures spacing between numbers and units is inconsistent. Supplemental Figure 6 uses “50mM” with no space and Figure 5 uses “30 ng” with a space. Please make spacing consistent throughout the Figures.

***Revised.***

11. Text color and font size are inconsistent through the Figures. Some text appears black while some text nearby appears a dark grey.

***Revised.***

12. Supplemental Figure 6 is missing “(“ from y-axis labels for panels E and D.

***Revised.***

13. Error bar presentation is not consistent across Figures or within them. In supplemental Figure 6 the lower half of error bars are present in panel B but not in Panel C.

***Corrected.***

14. Figure 4B, the colors in the bars bleed outside the lines in inconsistent ways.

***Revised.***

15. Capitalization and punctuations are used inconsistently throughout Figures. For example, the word “density” is lower case on the y-axis of 4E but uppercase on the y-axis of 5C. Also, in 5C “Dif” appears with a “.” while “Int” in 4E does not. Please correct consistency in capitalization and punctuation.

***Revised.***
16. Nomenclature varies within Figures. In supplemental Figure 6 panel B y-axis reads “(peak amplitute at 21 0 s)” while Panel D reads “Peak Apmplitude, t = 210s)”. It would make it easier for readers to interpret the paper if nomenclature was similar. Try making titles consistent when referring to similar things by using “at 210s” or “t=210s” instead of mixing within the same Figure.

***Revised.***
17. In supplemental Figure 6D “amplitude” is mis-spelled as “Apmplitude”. In the same Figure panel C “Accumulation” is mis-spelled as “Accumulationn”. In the same Figure panel B “amplitude” is mis-spelled as “amplitude”. Please check for spelling errors in the Figures.

***Revised.***
18. Usage of parenthesis and brackets is inconsistent throughout the Figures and within the same Figure. For example, supplemental Figure 5D uses “(3 min)” and “[3.5 min]” right next to each other. If the parenthesis and brackets do not represent something in specific please chance Figures to use one or the other consistently.

***Revised.***
19. Font usage is inconsistent throughout the Figures and within Figures. In supplemental Figure 6 the delta symbol has as different font in panel E and A.

***Revised.***
20. The symbol for hydroxyl groups in the Figures is not used consistently. In Figure 1C it appears as “•OH” with a circle in front of it, in supplemental Figure 1B it appears as “OH” with no symbol, and in supplemental Figure 3 it appears as with a square symbol in front of it.

***Corrected.***

21. Size bars are not consistently indicated in Figures. Figure 5 has no numbers or units denoting the size of the bars in the image while Figure 4 does. Also, the text on some of the bars in Figure 4F are too small to read. They should be replaced with vector graphics and scaled to be readable.

***Revised.***

22. In Figure 5G “++” label is missing from “Ca”.

***Revised.***
23. Abbreviation usage is inconsistent. Figure 3 uses both “hours” and “hrs”.

***Revised.***

24. Hash mark usage in plots is inconsistent. Figure 1A x-axis uses both log and linear scaling. Figure 3A axis has no marks but C does.

***Revised.***

25. Check spelling and grammar in Figures. Figure 1D uses “Cell lyse” as a label. Also see the same problem in the graphic of Figure 4A.

***Revised.***

26. Capitalization in nomenclature is inconsistent. Figure 4D uses “TAU” while panel C uses “Tau”.

***Revised.***

27. Figure 3D uses “mTau” and “Mouse Tau” in the same image. Nomenclature should be consistent.

***Revised.***

28. Figure 3D has size bars in the upper portions of the panels and not in the lower portions of the panel.

***Revised.***

29. Figures denote the origin of Tau in images as human or mouse. In Figure 4F there is no indicator of whether Tau is human or mouse.

***The nomenclature is explained in the Legend.***

30. Symbol usage in plots is inconsistent. Figure 4B uses upright triangles for OND (III) and panel E uses upright triangles for OND (I). Other OND symbols are also flipped.

***The different symbols are used to differentiate different experiments and they allow to link data obtained in Fig. 4B and Fig. 4E.***

PLOS authors have the option to publish the peer review history of their article ([what does this mean?](https://track.editorialmanager.com/CL0/https:%2F%2Fjournals.plos.org%2Fplospathogens%2Fs%2Feditorial-and-peer-review-process%23loc-peer-review-history/1/010f0195435c4eb8-d3331796-57b3-4be3-b6c4-0d08a2f924e8-000000/794KEy1NuCfbLN7faP8zy7SafCb3zjKdrPEAMnPrG6o=200)). If published, this will include your full peer review and any attached files.


If you choose “no”, your identity will remain anonymous but your review may still be made public.


Do you want your identity to be public for this peer review? For information about this choice, including consent withdrawal, please see our [Privacy Policy](https://track.editorialmanager.com/CL0/https:%2F%2Fwww.plos.org%2Fprivacy-policy/1/010f0195435c4eb8-d3331796-57b3-4be3-b6c4-0d08a2f924e8-000000/2EHgLBB8casNOgJ6-_yx7OILrtZm0wRfxwMkV6Z3uok=200).

Reviewer #1: No

Reviewer #2: No

Reviewer #3: No

[NOTE: If reviewer comments were submitted as an attachment file, they will be attached to this email and accessible via the submission site. Please log into your account, locate the manuscript record, and check for the action link "View Attachments". If this link does not appear, there are no attachment files.]

Figure resubmission:

While revising your submission, please upload your figure files to the Preflight Analysis and Conversion Engine (PACE) digital diagnostic tool, <https://pacev2.apexcovantage.com/>. PACE helps ensure that figures meet PLOS requirements. To use PACE, you must first register as a user. Registration is free. Then, login and navigate to the UPLOAD tab, where you will find detailed instructions on how to use the tool. If you encounter any issues or have any questions when using PACE, please email PLOS at [figures@plos.org](mailto:figures@plos.org). Please note that Supporting Information files do not need this step. If there are other versions of figure files still present in your submission file inventory at resubmission, please replace them with the PACE-processed versions.

Reproducibility:

To enhance the reproducibility of your results, we recommend that authors of applicable studies deposit laboratory protocols in protocols.io, where a protocol can be assigned its own identifier (DOI) such that it can be cited independently in the future. Additionally, PLOS ONE offers an option to publish peer-reviewed clinical study protocols. Read more information on sharing protocols at <https://plos.org/protocols?utm_medium=editorial-email&utm_source=authorletters&utm_campaign=protocols>

In compliance with data protection regulations, you may request that we remove your personal registration details at any time. [(Remove my information/details)](https://track.editorialmanager.com/CL0/https:%2F%2Fwww.editorialmanager.com%2Fppathogens%2Flogin.asp%3Fa=r/1/010f0195435c4eb8-d3331796-57b3-4be3-b6c4-0d08a2f924e8-000000/ooHdCxau3dtFmg9BxP6c9MYexXfvYzBqF0lezVugQak=200). Please contact the publication office if you have any questions.
